# Cardiac development and physiology are modulated by FGF2 in an isoform- and sex-specific manner

**DOI:** 10.1002/phy2.87

**Published:** 2013-09-17

**Authors:** Eyad Nusayr, Tom Doetschman

**Affiliations:** 1 Department of Cellular and Molecular Medicine, College of Medicine, The University of ArizonaTucson, Arizona; 2 BIO5 Institute, The University of ArizonaTucson, Arizona

**Keywords:** FGF2 isoforms, Echocardiography, sex, Physiology

## Abstract

The low-molecular-weight isoform (Lo) of fibroblast growth factor 2 (FGF2) has distinct functions from the high-molecular-weight isoforms (Hi) of FGF2 in the adult stressed heart. However, the specific roles of these isoforms in the unstressed heart were not examined. We investigated whether the FGF2 isoforms modulate cardiac development and physiology in isoform- and sex-specific manners. Young adult male and female mice that were deficient in either Hi FGF2 (Hi KO) or Lo FGF2 (Lo KO) underwent echocardiographic analysis and were compared to their wild-type (WT) counterparts. By comparison to WT cohorts, female Lo KO hearts display a 33% larger left ventricular (LV) volume and smaller LV mass and wall thickness. Mitral valve flow measurements from these hearts reveal that the early wave to atrial wave ratio (E/A) is higher, the deceleration time is 30% shorter and the mitral valve E-A velocity–time integral is reduced by 20% which is consistent with a restrictive filling pattern. The female Hi KO hearts do not demonstrate any significant abnormality. In male Hi KO mice the cardiac output from the LV is 33% greater and the fractional shortening is 29% greater, indicating enhanced systolic function, while in male Lo KO hearts we observe a smaller E/A ratio and a prolonged isovolumic relaxation time, consistent with an impaired relaxation filling pattern. We conclude that the developmental and physiological functions of FGF2 isoforms in the unstressed heart are isoform specific and nonredundant and that these roles are modulated by sex.

## Introduction

In the context of the heart, growth factors are critical for modulating cellular proliferation, differentiation and growth, extracellular matrix (ECM) deposition and composition, adhesion, migration, motility, apoptosis and responses to physiological and pathophysiological stimuli (Schneider and Parker 1991). Fibroblast growth factors (FGFs) comprise a large family of signaling molecules with various developmental as well as adult physiological and pathological functions (Itoh and Ornitz 2011). They are classically considered to be paracrine signaling molecules that modulate development. Moreover, there are a number of FGFs that act in an endocrine fashion with metabolic functions (Itoh and Ornitz 2011).

Fibroblast growth factor 2 (FGF2), a prototypic member of the FGF family, is encoded by a single gene but expressed in several isoforms due to alternative initiation of translation (Touriol et al. 2003). Eighteen kilodalton low-molecular-weight FGF2 (Lo FGF2) is translated from a conventional AUG start codon and consists of a 155 amino acid sequence common to all FGF2 isoforms. High-molecular-weight FGF2 (Hi FGF2) isoforms (20.5 & 21 kDa in mouse; 22, 22.5, 24, and 34 kDa in human) are generated by initiation of translation at upstream CUG codons in frame with the AUG (Prats et al. 1989).

Genetic overexpression or ablation of all isoforms (All KO) of the *Fgf2* gene in the mouse originally demonstrated roles for FGF2 in vascular tone control (Zhou et al. 1998), brain development (Dono et al. 1998), wound healing (Ortega et al. 1998), and blood pressure regulation (Dono et al. 1998). With respect to the heart we have shown that FGF2 mediates both hypertrophy (Schultz et al. 1999; House et al. 2010) and protection from ischemia–reperfusion (I/R) injury (House et al. 2005).

The first indication that Hi and Lo FGF2 must have differential functions was that their protein expression levels relative to each other are tissue specific and independent of the genomic location of their transgenes (Coffin et al. 1995). The differential subcellular localization and trafficking of FGF2 isoforms were additional indications that these isoforms have distinct, nonredundant functions. While Hi FGF2s are mainly intracellular, Lo FGF2 is released from cells to signal via its interaction with high-affinity transmembrane FGF receptors (FGFR1-4) in a paracrine or autocrine manner (Yu et al. 2007; Liao et al. 2010). Several in vitro studies have explored the isoform-specific functions of FGF2 (Yu et al. 2007).

To explore the isoform-specific functions of FGF2 in vivo we generated two mouse strains, one with a deleted Lo FGF2 isoform (Lo KO) (Garmy-Susini et al. 2004) and the other with deleted Hi FGF2 isoforms (Hi KO) (Azhar et al. 2009). This was done using the Tag & Exchange procedure (Askew et al. 1993) that leaves no residual loxP sites that might interfere with the regulatory information and non-FGF2-related reading frames embedded within the first intron of the gene. These mice demonstrate differential functions of FGF2 isoforms. For example, Hi but not Lo FGF2 isoforms mediate endothelial cell migration and proliferation in an intracrine fashion (Garmy-Susini et al. 2004) while Lo FGF2 modulates bone density (Xiao et al. 2009).

In the context of the heart it was discovered that it is the Lo FGF2 alone that mediates the protective effect following I/R injury (Liao et al. 2007) while the Hi FGF2 isoforms are detrimental (Liao et al. 2010). These I/R injury studies provided insight into the isoform-specific functions of FGF2 in the stressed heart; however, whether these isoforms have a role in the unstressed heart that would predispose them to the phenotypes observed in the I/R models was not characterized. The objective of this study was to determine the isoform-specific functions of FGF2 in the unstressed heart.

Transthoracic echocardiography (Echo) is a reliable method of discerning changes in cardiac geometry and systolic and diastolic function in mice (Tanaka et al. 1996). To examine the role of FGF2 isoforms in the unstressed heart we performed Echo analysis on young adult male and female Hi and Lo KO mice. This is the first report on the effect of FGF2-isoform deletion in producing distinct, sex-specific functional, and morphological phenotypes, thus providing evidence that Lo and Hi FGF2 are mechanistically distinct in their interaction with sex to modulate cardiac development and physiology.

## Material and Methods

### Animals

Wild-type (WT), Lo KO (*Fgf2*^*tm2Doe*^/J), and Hi KO (*Fgf2*^*m3Doe*^/J) FGF2 mice were used for this study. These mice were generated as described previously (Garmy-Susini et al. 2004; Azhar et al. 2009) and bred on a mixed Black Swiss (50%)/129 (50%) background. The mice were 8–12 weeks of age and were housed in an specific pathogen free vivarium at the University of Arizona that is *Helicobacter* and norovirus free. Animals in the facility are housed in microisolator cages in ventilated cage racks with automatic watering. This study was approved by IACUC #09-037 entitled “Cardiac Hypertrophy Studies.” Genotyping of these mice was performed by the University of Arizona Genetics Core (UAGC) in the University of Arizona's Arizona Research Laboratory (ARL). Five to 10 (Tables 1 and 2) mice from each sex/genotype group were used for each data point.

**Table 1:** Female gravimetric and geometric measurements

				WT vs. Lo KO	WT vs. Hi KO	Lo Ko vs. Hi KO
	WT	Lo KO	Hi KO	*P*-value	*P*-value	*P*-value
*n*	5	6	8			
Body mass (g)	22 ± 0.6	25.3 ± 1.3^Δ^	21.5 ± 0.5	0.048	NS	0.027
IVS;d	0.64 ± 0.07	0.50 ± 0.03	0.59 ± 0.01	NS	NS	0.021
IVS;s	0.75 ± 0.05	0.64 ± 0.07	0.79 ± 0.05	NS	NS	NS
LVPW;d	0.65 ± 0.03	0.49 ± 0.02^Δ^	0.62 ± 0.05	0.003	NS	0.038
LVPW;s	0.91 ± 0.04	0.67 ± 0.05^Δ^	0.79 ± 0.06	0.006	NS	NS
LVID;d	3.58 ± 0.11	4.06 ± 0.07^Δ^	3.68 ± 0.10	0.007	NS	0.012
LVID;s	2.57 ± 0.15	3.00 ± 0.12	2.76 ± 0.16	NS	NS	NS

Values are means ± SEM. Dimensions are in millimeters. *n*, no. of mice; IVS;d and IVS;s, Interventricular septum thickness in diastole and systole, respectively; LVPW;d and LVPW;s, Left ventricular posterior wall thickness in diastole and systole, respectively; LVID;d and LVID;s, Left ventricular internal dimension in diastole and systole, respectively; NS, not significant. ^Δ^Statistical significance versus WT.

**Table 2:** Male gravimetric and geometric measurements

				WT vs Lo KO	WT vs. Hi KO	Lo Ko vs. Hi KO
	WT	Lo KO	Hi KO	*P*-value	*P*-value	*P*-value
*n*	10	8	10			
Body mass (g)	29.8 ± 1.6	31.4 ± 1.4	26.6 ± 1.0	NS	NS	0.015
IVS;d	0.68 ± 0.04	0.61 ± 0.05	0.71 ± 0.03	NS	NS	NS
IVS;s	0.86 ± 0.04	0.78 ± 0.04	0.89 ± 0.04	NS	NS	NS
LVPW;d	0.59 ± 0.02	0.61 ± 0.06	0.56 ± 0.03	NS	NS	NS
LVPW;s	0.83 ± 0.05	0.85 ± 0.10	0.84 ± 0.06	NS	NS	NS
LVID;d	4.27 ± 0.10	4.29 ± 0.08	4.06 ± 0.08	NS	NS	NS
LVID;s	3.25 ± 0.11	3.24 ± 0.10	2.78 ± 0.12*	NS	0.014	0.012

Values are means ± SEM. Dimensions are in millimeters. *n*, no. of mice; IVS;d and IVS;s, Interventricular septum thickness in diastole and systole, respectively; LVPW;d and LVPW;s, Left ventricular posterior wall thickness in diastole and systole, respectively; LVID;d and LVID;s, Left ventricular internal dimension in diastole and systole, respectively; NS, not significant.

* Statistical significance versus WT.

### Echocardiography

Echo was performed on a Vevo 770 (Visual Sonics, Toronto, Canada), using the specially adapted 707B scan head. Before imaging, the mice were anesthetized with 3% isoflurane mixed with 100% oxygen, and anesthesia was thereafter maintained with 1.5% isoflurane. The mice were depilated as required for imaging and placed on a 37°C heated platform throughout the imaging procedure. Echo measurements were taken from at least three different cardiac cycles for each mouse.

### Geometric systolic and diastolic measurements

**Figure 1:**
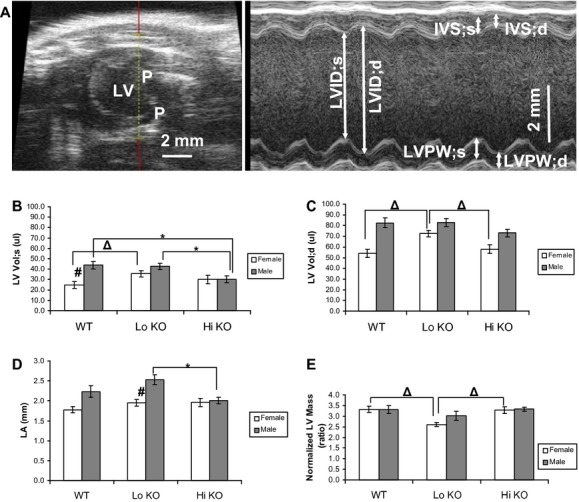
Assessment of cardiac geometry and gravimetry in the left ventricle of female (white bar) and male (gray bar) wild-type (WT), low-molecular-weight FGF2 knockout (Lo KO) and high-molecular-weight FGF2 knockout (Hi KO) hearts. Note the larger left ventricular volume and smaller mass of female Lo KO hearts which is consistent with dilated cardiomyopathy. (A) Representative short-axis view image (Left) showing Left Ventricle (LV) and Papillary muscles (P) and related M-mode image (right) showing interventricular septal thickness in diastole and systole (IVS;d and IVS;s, respectively), LV posterior wall thickness in diastole and systole (LVPW;d and LVPW;s, respectively). LV internal dimension in diastole and systole (LVID;d and LVID;s, respectively). (B) LV volume in systole (LV Vol;s). (C) LV volume in diastole (LV Vol;d). (D) Left atrial dimension (LA). (E) LV mass normalized to body weight. (Δ) Statistical significance across female genotypes. (*) Statistical significance across male genotypes. (#) Statistical significance across sex for same genotype. Each bar represents data from 5 to 10 animals; graphs are presented as means ± SEM for each genotype/sex and significance noted when *P* < 0.05.

M-mode imaging from the short axis (midventricular plane) of the left ventricle (LV), using the papillary muscles for reference (Fig. 1A), was used to obtain measurements of interventricular septum thickness in diastole and systole (IVS;d and IVS;s, respectively), Left ventricular posterior wall thickness in diastole and systole (LVPW;d and LVPW;s, respectively) and left ventricular internal dimension in diastole and systole (LVID;d and LVID;s, respectively). M-mode imaging (not shown) was also used to obtain left atrial dimension (LA) as described previously (Finsen et al. 2005). Long-axis (horizontal apex to outflow tract plane)-B-mode imaging (not shown) was used to acquire heart rate (HR), endocardial area in systole and diastole and the endocardial major in systole and diastole (distance from aortic valve to apex in systole and diastole, respectively). For measuring systolic flow parameters pulse wave (PW)-Doppler interrogation was used in the aortic arch and the pulmonary artery (Fig. 3A) thus obtaining the mean flow velocity of the aortic arch (Ao;Vel), the aortic arch velocity–time integral (Ao;VTI), and the mean flow Velocity of the Pulmonary artery (Pul;Vel). PW-Doppler was also used to measure mitral valve (MV) flow parameters in the four-chamber view (Fig. 4A), acquiring the early wave (E) and atrial wave (A) velocities, the mitral valve E-deceleration time (MV;Dec), the isovolumic relaxation time (IVRT), the mitral valve E-A velocity–time integral (MV;VTI), and the mean flow velocity (MV;Vel).

### Calculations

Based on the direct Echo measurements we used the Vevo 770 software (v3.0.0) to calculate dependent Echo parameters of the heart. The following calculations were used: LV volume in systole (LV Vol;s) = (7.0/(2.4 + LVID;s)) × LVID;s^3^; LV volume in diastole (LV Vol;d) = (7.0/(2.4 + LVID;d)) × LVID;d^3^; LV Mass = 1.053 × ((LVID;d + LVPW;d + IVS;d)^3^ – LVID;d^3^), the LV mass was then normalized to body mass; endocardial volume in diastole = (4π/3)(endocardial major in diastole/2)(Endocardial area in diastole/π(endocardial major in diastole/2))^2^; endocardial volume in systole = (4π/3)(endocardial major in systole/2)(endocardial area in systole/π(endocardial major in systole/2))^2^; stroke volume (SV) = Endocardial Volume in diastole − Endocardial Volume in systole; cardiac output (CO) = HR × Endocardial SV/2; LV Fractional Shortening (FS)% = 100 × ((LVID;d − LVID;s)/LVID;d); mitral valve E to A ratio = E/A; mitral valve peak pressure Gradient (MV;Grad) = (4 × (Mitral valve peak velocity)^2^)/10^6^. The velocity–time Integral (VTI) for any PW-Doppler velocity measurements was calculated by integrating over the time interval.

### Statistical analysis

Two-sided student *t*-test with unequal variance was used to evaluate the data. A difference was considered significant when *P* < 0.05. In the panels of barographs the height of the bar represents the mean while the error bar represents the standard error of the mean.

## Results

### Differences in cardiac development in Hi and Lo KO animals are sex and isoform specific

Echo LV mass is a parameter for determination of LV mass that was extrapolated from systematic analysis of the relationship between the antemortem LV Echo and postmortem anatomic LV Mass (Devereux and Reichek 1977). In heart disease, increased LV mass is a strong predictor of an adverse prognosis (Apstein and Lorell 1988). Using the two-dimensional M-mode (Fig. 1A), we measured the internal dimension of the LV in systole and diastole and its wall thicknesses (Tables 1 and 2). These measurements were used to calculate the LV volume in systole and diastole (Fig. 1B and C) and the LV mass which was normalized to body mass (Fig. 1E). Male Hi KO heart LVs display a significantly smaller volume in systole (32% less than WT cohorts) but not diastole. Female Lo KO heart LVs, however, display an increased volume in both systole (44%) and diastole (33%) relative to WT cohorts. Interestingly, when the Echo-calculated LV mass is normalized to body mass, female Lo KO hearts are smaller than either the WT controls or the Hi KO female hearts (Fig. 1E).

We also used M-Mode to obtain the LA dimension (not shown). LA dimension can be indicative of aortic stenosis and left atrial enlargement (Chandraratna et al. 1982) and an increase in LA dimension accompanies dilated cardiomyopathy, LV hypertrophy, diastolic dysfunction or left atrial abnormality (Tsang et al. 2002). Male Lo KO hearts have an LA dimension larger than that of male Hi KO hearts (Fig. 1D).

Female Lo KO heart LVs present with a 25% thinner LVPW;d and a 14% larger LVID;d relative to WT cohorts as measured by M-mode imaging (Table 1). In comparison, male mutant mice display no significant differences in these parameters (Table 2). In males, Hi KO mice have 15% smaller body weight when compared to their Lo KO counterparts (Table 2) and WT mice are not significantly different than Lo KO mice. In females, Lo KO mice have 15% larger body mass over both WT and Hi KO mice (Table 1).

A larger diastolic LV volume and thinner LV walls to volume ratio are correlated clinically with dilated cardiomyopathy (Kuroda et al. 1989). Here, we show that only the female Lo KO heart presents with both of these abnormalities; the LV of male Lo KO hearts has normal volume and weight. The LV dilation, smaller LV mass, and heavier body weight of female Lo KO mice suggest that Lo FGF2 has a greater role in female heart and body growth and geometry than in males.

Sex comparisons reveal that LV systolic volume is larger in WT males than WT females. This sex difference is absent from the Hi KO group suggesting that Hi FGF2 is important for the normalcy of LV systolic volume only in males (Fig. 1B). Furthermore, LA dimension is larger in male WT and Lo KO animals than in females (Fig. 1D), but it is the same in male Hi KO hearts denoting a role for Hi FGF2 in determining LA dimension in males.

### Systolic functional differences in Hi and Lo KO hearts are sex and isoform specific

Echo parameters of systolic function include SV, CO, and FS. SV is the volume of blood that the LV pumps in one beat, and it is used to calculate CO which is the volume of blood being pumped by the LV in a minute. FS is the fraction of LV diastolic dimension that is lost in systole (Rottman et al. 2007).

**Figure 2:**
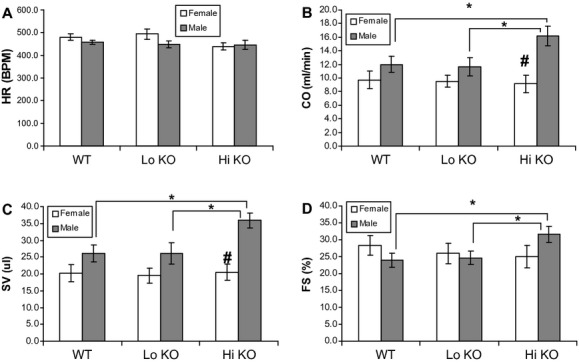
Assessment of cardiac systolic function in the left ventricle of female (white bar) and male (gray bar) WT, Lo KO, and Hi KO hearts. Note the greater cardiac output of male Hi KO hearts which results from a larger stroke volume but not a higher heart rate. (A) Heart rate. (B) Cardiac output (CO). (C) Stroke volume (SV). (D) Fractional shortening (FS). (*) Statistical significance across male genotypes. (#) Statistical significance across sex for same genotype. Each bar represents data from 5 to 10 animals, Graphs are presented as means ± SEM for each genotype/sex and significance noted when *P* < 0.05.

Using the measurements from B-mode imaging (not shown), we obtained CO and SV. M-mode imaging (Fig. 1A) was used to calculate FS. Although HR is not significantly different across groups (Fig. 2A), male Hi KO hearts display a greater CO (33%), larger SV (38%), and higher FS (29%) when compared to WT cohorts (Fig. 2B–D). Hence, the larger CO measured in Hi KO male hearts is not a result of a higher HR, but rather a significantly larger SV and FS. Female Lo and Hi KO hearts do not present with any significant differences in Echo systolic parameters when compared to their WT cohorts (Fig. 2B–D).

In sex comparisons the higher CO, SV, and FS exhibited by male Hi KO hearts are also significantly higher than the female cohorts (Fig. 2B–D). This sex difference is absent from both the Lo KO and WT groups indicating that Hi FGF2 is only necessary in males for normalcy of LV systolic function.

### Systolic flow differences in Hi and Lo KO hearts are sex and isoform specific

**Figure 3:**
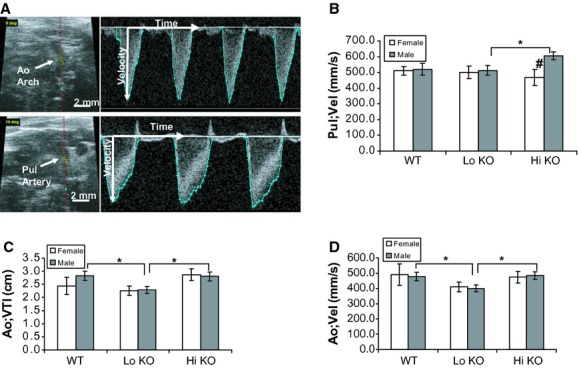
Assessment of cardiac systolic flow through Pulse wave-Doppler analysis in female (white bar) and male (gray bar) WT, Lo KO, and Hi KO hearts. Note the smaller aortic flow parameters of male Lo KO hearts which is consistent with a larger aortic cross-sectional area as stoke volume is preserved. (A) Representative two-dimensional images showing aortic arch (Ao arch) and pulmonary artery (Pul artery) in upper and lower left panels, respectively, and respective PW-Doppler images used to obtain measurements. (B) Flow velocity in Pul artery (Pul;Vel). (C) Aortic velocity–time integral (Ao;VTI) from the aortic arch flow. (D) Aortic flow mean velocity (Ao;Vel). (*) Statistical significance across male genotypes. (#) Statistical significance across sex for same genotype. Each bar represents data from 5 to 10 animals; graphs are presented as means ± SEM for each genotype/sex and significance noted when *P* < 0.05.

Using PW-Doppler (Fig. 3A) we measured Ao;VTI, Pul;Vel, and Ao;Vel which are systolic flow parameters of both the right and left side of the heart. Male Hi KO hearts exhibit a 16% higher Pul;Vel (Fig. 3B) while male Lo KO hearts display a lower Ao;Vel (18%) and Ao;VTI (19%)(Fig. 3C and D). The female hearts on the other hand show no significant differences in these measurements between experimental groups.

Thus, the observation that Hi KO male hearts have enhanced systolic function is also supported by the systolic PW-Doppler data that show a higher velocity of pulmonary artery flow (Gentile et al. 1993). Curiously, the Ao;VTI in Hi KO male hearts was not significantly different than in the WT controls. Multiplying the VTI by the cross-sectional area (CSA) of the flow tract gives the SV in that tract; SV = CSA×VTI. The SV of the LV in these hearts is higher, indicating a larger CSA of the aorta (Evangelista et al. 2010). On the other hand, the male Lo KO hearts presented smaller aortic flow parameters; a smaller Ao;VTI value, and a slower Ao;Vel. As the SV of these hearts is comparable to WT cohorts then the plausible cause of these smaller aortic flow parameters is a wider, more compliant aorta (Evangelista et al. 2010).

Comparing the hearts by sex reveals that Pul;Vel is only significantly higher in Hi KO males versus female cohorts (Fig. 3B). This sex difference is absent in the Lo KO and WT groups, indicating that Hi FGF2 is important only in males for normal right-sided systolic flow velocity.

### Diastolic flow differences in Hi and Lo KO hearts are sex and isoform specific

**Figure 4:**
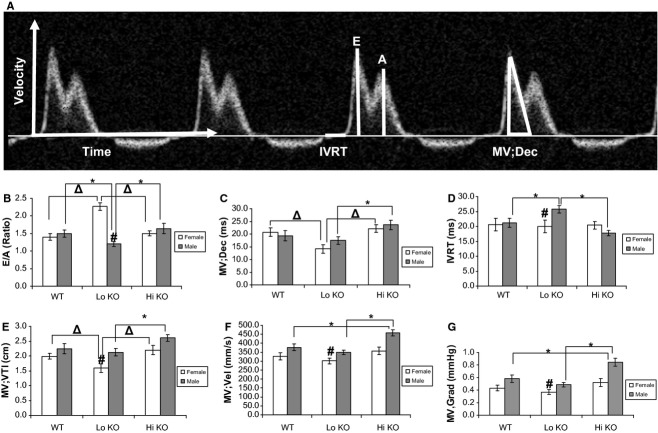
Assessment of cardiac diastolic flow through pulse wave-Doppler analysis in female (white bar) and male (gray bar) WT, Lo KO, and Hi KO hearts. Note that the female Lo KO heart displays a large E to A ratio and a shorter E-wave deceleration time which is consistent with a restrictive filling pattern, while the male Lo KO heart displays a small E to A ratio and a longer isovolumic relaxation time which is consistent with an impaired relaxation filling pattern. (A) Representative PW-Doppler image of the flow through the mitral valve showing early wave (E), atrial wave (A), isovolumic relaxation time (IVRT), and mitral valve E-wave deceleration time (MV;Dec) measurements. (B) Early wave to atrial wave ratio (E/A). (C) MV;Dec. (D) IVRT. (E) Mitral valve E-A velocity–time integral (MV;VTI). (F) Mitral valve flow mean velocity (MV;Vel). (G) Mitral valve flow mean pressure gradient (MV;Grad). (Δ) Statistical significance across female genotypes. (*) Statistical significance across male genotypes. (#) Statistical significance across sex for same genotype. Each bar represents data from 5 to 10 animals; graphs are presented as means ± SEM for each genotype/sex, and significance is noted when *P* < 0.05.

PW-Doppler measurements of blood-flow velocity across the mitral valve are used to assess LV filling noninvasively (Oh et al. 1997). The pressure gradient between the LA and the LV in addition to ventricular suction determine early inflow velocity through the opening of the mitral valve (Garcia et al. 1998). PW-Doppler measurements at the MV were used to obtain the E/A ratio, the MV;Dec, IVRT, MV;VTI, MV;Vel, and MV;Grad (Fig. 4A). Classically, there are four distinct E/A ratio patterns (normal, delayed relaxation, pseudonormal, restrictive), and these can be discerned if viewed within the context of other available Echo parameters (Vitarelli and Gheorghiade 1998). MV;Dec is a measure of how rapidly early diastolic filling stops. When filling pressures are high and chamber stiffness increases, early mitral filling will end abruptly, resulting in a shortened MV;Dec and a restrictive filling pattern (Garcia et al. 1998). MV;Dec is represented by the time interval between the E peak and a point on the baseline where the descending limb crosses the baseline (Fig. 4A). The IVRT is the interval between aortic valve closure and the onset of LV filling at the MV opening (Fig. 4A). In clinical hypertension abnormal IVRT correlates with both increased LV mass and decreased rapid LV filling (Smith et al. 1987). Prolonged IVRT is also correlated with abnormal diastolic function (Vitarelli and Gheorghiade 1998). The MV;VTI area represents the diastolic filling of the LV due to the rapid filling phase and atrial contraction. MV;VTI is used to calculate flow volume (Gentile et al. 1993). The conservation of flow is represented by the continuity equation and allows us to use Echo to determine the CSA of MV (Evangelista et al. 2010).

The results indicate that normal diastolic function in male and female hearts depends more on Lo FGF2 than it does on Hi FGF2. Here, we show that the E/A ratio deviates from WT in both male and female Lo KO hearts albeit in opposite directions; female Lo KO hearts display a significantly larger E/A ratio, while male Lo KO hearts have a lower E/A ratio relative to WT cohorts (Fig. 4B). The shortened MV;Dec in female Lo KO hearts (30% less than WT cohort) (Fig. 4C) is consistent with myocardial stiffness; and when combined with the larger E/A ratio, which is a result of increased peak E-wave velocity with a diminutive A wave (data not shown), it is indicative of a restrictive filling pattern. The prolonged IVRT in male Lo KO hearts (25% more than WT cohorts) (Fig. 4D) when combined with the smaller E/A ratio, which is a result of a lower E wave and a higher A-wave velocity (data not shown), is consistent with a slower decline in LV pressure and the impaired relaxation filling pattern (Vitarelli and Gheorghiade 1998).

Female Lo KO hearts show a reduced MV;VTI (20% less than WT cohorts) (Fig. 4E) which when combined with the normal SV observed in these hearts suggests a larger MV CSA. Consistent with the enhanced systolic function, male Hi KO hearts display a 21% increase in MV;Vel and a 44% higher MV;Grad (Fig. 4F and G).

When comparing across sex we find that the E/A ratio is significantly higher in female Lo KO hearts than their male cohorts; this sex difference is absent from both the WT and Hi KO groups (Fig. 4B). On the other hand, the IVRT, the MV;VTI, the MV; Vel, and the MV;Grad are all higher in the male Lo KO hearts than in the female cohorts (Fig. 4D–G). This sex difference is absent from both WT and Hi KO groups indicating an important, sex-specific role for Lo FGF2 in determining diastolic function.

## Discussion

This study provides novel evidence on the interaction of sex and endogenous FGF2 isoforms as modulators of cardiac development and physiology. Lo FGF2 signaling is necessary in the female heart only for normal myocardial growth while in both male and female hearts Lo FGF2 signaling is required for normal diastolic function. On the other hand, Hi FGF2 is necessary only in male hearts for modulating systolic function and does not display a physiological role in female hearts. Hence, we demonstrate that Lo FGF2 and Hi FGF2 have nonredundant roles in cardiac development and physiology which are sex specific.

Sex differences in cardiac phenotype are well documented in both humans and animal models. With some exceptions, estrogen is associated with a cardioprotective effect while male hormones are considered to be detrimental (Du 2004). To date, there is no report on the interaction of FGF2 and sex in cardiac development and physiology. Our Lo KO and Hi KO hearts display a sexual dimorphism that is not present in the WT hearts, providing evidence that FGF2 isoforms have sex-specific physiological functions.

FGF2 is expressed early in the developing embryonic heart. This expression is spatially and temporally regulated (Liao et al. 2009). Hence, it is intuitive to assume that FGF2 plays an important role in heart development and physiology. Earlier studies showed that genetically manipulated mice that either overexpress FGF2 or have a null deletion of FGF2 display developmental abnormalities that affect the nervous system, bone morphology, and vascular tone (Coffin et al. 1995; Dono et al. 1998; Zhou et al. 1998). In the heart there were no significant phenotypes at baseline conditions. However, as Lo FGF2 and Hi FGF2 can be antithetical, the deletion of one isoform in mice while maintaining the other would produce a more measurable biological effect than deleting both groups of isoforms. With the advent of the Lo KO and the Hi KO mouse strains we have the tools necessary to understand the in vivo role of endogenous FGF2 isoforms in cardiac development and physiology.

The female Lo KO heart is interesting in that it has a smaller mass at baseline while the cardiomyocyte cross-sectional area is normal. This is reminiscent of the smaller heart that results from IGF-I receptor deficiency (Holzenberger et al. 2001). FGF2 is also a potent regulator of cardiac myocyte and fibroblast proliferation in vitro (Watkins et al. 1998; Dealmeida and Sedmera 2009) indicating that Lo FGF2 in females plays a similar developmental role to IGF-I. Whether this smaller mass is a result of less cardiomyocytes, nonmyocyte cells (NMCs), or ECM remains to be examined. Of note is that in developing hearts FGF2 is highly expressed in the compact myocardium, the epicardium, and epicardial-derived cells (Liao et al. 2009). The epicardium is a significant source of NMCs in heart development (Lavine and Ornitz 2008), and it is likely that Lo FGF2 signaling is necessary for modulating NMCs (mostly fibroblasts) and their functions in the developing female heart. A previous study demonstrated that estrogen induces cell migration through upregulation of FGFR1 expression and this induction is mediated by Hi FGF2 (Garmy-Susini et al. 2004). The results of this study demonstrate that Lo FGF2 is required for normal cardiac development which likely signals through FGFR1 thereby eliciting mitotic pathways. In male Lo KO hearts we observed a larger LA dimension when compared to Hi KO hearts (Fig. 1D). Whether this increase in LA dimension is developmental or a result of the observed pathological filling pattern remains to be examined. No association between FGF2 signaling and male sex hormones has been reported.

Lo KO and Hi KO mice were previously used in I/R studies to reveal differential pathophysiological functions of FGF2 isoforms in the heart (Montero et al. 2000; Garmy-Susini et al. 2004; Liao et al. 2007, 2010), and they revealed that Hi and Lo FGF2 are part of the I/R stress response. Our results are from nonstressed hearts and reveal novel developmental and physiological roles of FGF2 isoforms that may predispose the isoform-deficient hearts to differential responses to cardiac stress. For example, in the I/R model, the Hi KO heart was protected from injury while the Lo KO heart was more susceptible. Since at baseline the Lo KO presents with diastolic dysfunction, it is intuitive that the under stress from I/R the injury would be more severe. However, the I/R studies did not present sex-specific results so it would be of interest to evaluate the data from those studies in light of the results from this report. Furthermore, it is recommended that any future study of FGF2 isoforms and the cardiovascular system be analyzed according to sex.

Although endogenous FGF2 clearly acts locally in heart tissue (Pellieux et al. 2001; House et al. 2005, 2007), that does not exclude a systemic effect on heart physiology as can be seen in the male Hi KO mice that exhibit a smaller body mass when compared to Lo KO males, or the heavier body weight of Lo KO females when compared to both WT and Hi KO females. This could in part result from the role of FGF2 in modulating bone development and vascular tone (Zhou et al. 1998; Montero et al. 2000).

Hi FGF2 is essential for normal systolic function in male but not in female hearts. The increased SV and FS in male Hi KO hearts (Fig. 2C and D) could be a result of increased contractility but SV and FS are also dependent on preload and afterload (Colan et al. 1984). Clinically, an increase in CO and SV with preserved HR is consistent with isolated systolic hypertension (McEniery 2005), and the absence of Hi FGF2 could be causing abnormal aortic morphology and endothelial dysfunction (Wallace 2007). This is especially plausible as Hi FGF2 is implicated in endothelial proliferation and migration (Garmy-Susini et al. 2004). Whether the cause of the increased FS and SV is enhanced contractility or changes in cardiac load remains to be examined. However, the results of this study suggest that the aortas of these mice are wider or more compliant which correlates with decreased afterload. Moreover, the mitral valve flow parameters are higher in male Hi KO hearts (Fig. 4E–G) which correlates with increased preload. The higher CO observed in these hearts could be the result of a combination of both increased preload and decreased afterload rather than increased contractility. However, this would need to be verified via direct measurements of preload and afterload and blood pressure measurements. The dilated aorta is also a feature of male Lo KO mice, suggesting that both Hi and Lo FGF2 are required for normal aortic development. An examination of the molecular and mechanical properties of the aorta may reveal the role of FGF2 isoforms in its development.

Doppler interrogation of diastolic flow further elucidated a sex- and isoform-specific phenotype where male KO hearts display an impaired relaxation pattern. At the cardiomyocyte level, relaxation involves the removal of Ca^2+^ from troponin-C through sarcoplasmic reticulum uptake and subsequent detachment of actin–myosin cross-bridges (Yellin et al. 1990). Ca^2+^ dysregulation is a possible cause for the impaired relaxation observed in the male Lo KO hearts. Diastolic flow data from the female Lo KO hearts indicates a stiff LV. Clinically, stiffness is a part of diastolic dysfunction and is usually the consequence of pathological changes in either the ECM or the cardiomyocytes. Increased collagen synthesis, decreased degradation or increased cross-linking all can contribute to LV stiffness (Ahmed et al. 2006). Intrinsic cardiomyocyte stiffness may arise from changes to the giant elastic protein Titin, cross-bridge detachment, and Ca^2+^ uptake (Neagoe et al. 2003). Matricellular proteins are ECM proteins that regulate cell–matrix interactions and cell function. As they interact with both the ECM and the cardiomyocytes they could potentially play an important role in diastolic dysfunction (Schellings et al. 2004). FGF2 is expressed in both cardiomyocytes and nonmyocyte cells (NMCs) of the myocardium (data not shown), the latter being responsible for ECM production and remodeling (Adler et al. 1981) and it is likely that FGF2 isoforms have differential functions in those two populations of cells. Hence, the deletion of Lo FGF2 in females could dysregulate the ECM and intrinsic cardiomyocyte elasticity causing stiffness, while deletion of Lo FGF2 in males could result in dysregulation of the contractile apparatus and consequent impaired relaxation.

**Figure 5:**
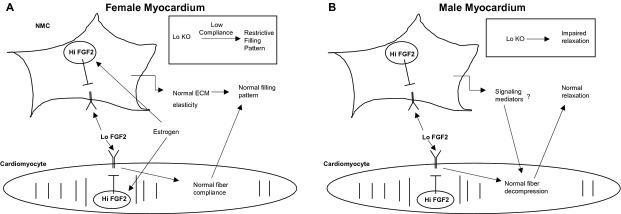
Model of the physiologic role of FGF2 isoforms. (A) In the female myocardium low-molecular-weight FGF2 (Lo FGF2) is required for physiologic myocardial compliance which results in normal filling pattern of the LV. This physiological role of Lo FGF2 is dependent on the gene-modulatory function of estrogen and is negatively regulated by high-molecular-weight FGF2 (Hi FGF2). Ablation of Lo FGF2 results in a dysregulation of nonmyocyte cells (NMCs) and subsequent loss of extracellular matrix (ECM) elasticity and fiber stiffness manifesting in a restrictive filling pattern. (B) In the male cardiomyocyte Lo FGF2 is required for physiologic fiber decompression. Hi FGF2 negatively regulates Lo FGF2 signaling while ablation of Lo FGF2 causes a delay in fiber decompression and manifests in an impaired relaxation pattern. Lo FGF2 signaling in NMCs contributes to normal fiber decompression through unidentified signaling mediators. (↑) Stimulatory signal; (⊥) inhibitory signal.

In light of the results of this study, a model of the role of FGF2 isoforms in cardiac diastolic physiology emerges (Fig. 5). In the female cardiomyocyte Lo FGF2 signaling through FGFR leads to a physiologic compliant muscle fiber while in NMCs it leads to production of ECM with normal elasticity. This role of Lo FGF2 is antagonized by Hi FGF2 at the receptor level and possibly other downstream effectors. In the absence of Lo FGF2 the Lo KO heart is stiffer and displays a restrictive filling pattern (Fig. 5A). Estrogen modulates gene expression and signaling pathways in both cardiomyocytes and NMCs to a profile that renders them amiable to Lo FGF2 as a regulator of fiber compliance. In the male cardiomyocyte Lo FGF2 signaling contributes to physiological muscle fiber decompression and normal relaxation. In the absence of Lo FGF2 the Lo KO cardiomyocyte has delayed fiber decompression and the Lo KO heart displays impaired relaxation (Fig. 5B). Due to the intimate signaling relationship between NMCs and cardiomyocytes, we also propose that Lo FGF2 signaling in male NMCs causes an induction of unidentified signaling mediators that contribute to Lo FGF2-dependent normal fiber compression. In short, we identify key molecules at the apex of two molecular mechanisms that modulate cardiac physiology and development; one involving Hi FGF2 and the other Lo FGF2.

The results of this study demonstrate that Lo KO and Hi KO mice are useful tools for studying diastolic dysfunction. The importance of understanding diastolic dysfunction stems from the prognosis of diastolic heart failure that accompanies it. Almost half of patients diagnosed with heart failure are classified under the diastolic heart failure category (Zile and Brutsaert 2002). Unlike systolic heart failure, what characterizes diastolic heart failure is a preserved systolic property with increased LV stiffness and abnormal relaxation (Zile et al. 2005). The danger of diastolic dysfunction is that it is clinically silent, and the diagnosis is based upon findings of abnormal LV filling patterns (Aurigemma and Gaasch 2004). Although FGF2 isoforms are not known to be separately expressed under physiological or pathological conditions, the results presented indicate a therapeutic benefit in manipulating FGF2 isoform signaling. As Lo FGF2 protects the heart from pathophysiological changes in diastolic function in both males and females, inducing Lo FGF2 signaling in patients with diastolic dysfunction would serve to ameliorate or even reverse this dysfunction. An additional benefit could also be achieved by inhibiting Hi FGF2 signaling as it functions antithetically to Lo FGF2 at the receptor level (Liao et al. 2010) and possibly at the transcription level.

Previous studies were inconclusive on whether FGF2 modulates cardiac growth and development. Our laboratory showed that all KO mice develop normally and have normal cardiac structure and mass (Zhou et al. 1998; Schultz et al. 1999). Only when these hearts where stressed was an FGF2-dependent phenotype realized (Schultz et al. 1999; House et al. 2010). As Hi FGF2 and Lo FGF2 display antithetical properties in the heart it follows that deleting both of them could have a more subtle phenotype than deleting one and leaving the other intact.

Even though we reveal a sex-specific role of FGF2 isoforms in cardiac physiology and development, the pathways through which the isoforms signal to modulate cardiac physiology and development are not fully understood. Of special note is the lack of a detailed description of Hi FGF2 signaling and its interaction Lo FGF2 or with its effectors downstream. The interaction of estrogens and androgens with FGF2 isoforms to produce the sex-specific phenotypes observed here also remains to be investigated. In the context of the heart alone, how the isoforms modulate physiological hypertrophy or myocardial pathological remodeling, including ECM composition and cross-talk between the cellular constituents of the myocardium, are questions with possible clinical significance. With our Lo and Hi KO mice we can begin to address these and other questions.

## References

[b1] Adler CP, Ringlage WP, Bohm N (1981). DNA content and cell number in heart and liver of children. Comparable biochemical, cytophotometric and histological investigations (author's transl). Pathol. Res. Pract.

[b2] Ahmed SH, Clark LL, Pennington WR, Webb CS, Bonnema DD, Leonardi AH (2006). Matrix metalloproteinases/tissue inhibitors of metalloproteinases: relationship between changes in proteolytic determinants of matrix composition and structural, functional, and clinical manifestations of hypertensive heart disease. Circulation.

[b3] Apstein CS, Lorell BH (1988). The physiological basis of left ventricular diastolic dysfunction. J. Card. Surg.

[b4] Askew GR, Doetschman T, Lingrel JB (1993). Site-directed point mutations in embryonic stem cells: a gene-targeting tag-and-exchange strategy. Mol. Cell. Biol.

[b5] Aurigemma GP, Gaasch WH (2004). Clinical practice. Diastolic heart failure. N. Engl. J. Med.

[b6] Azhar M, Yin M, Zhou M, Li H, Mustafa M, Nusayr E (2009). Gene targeted ablation of high molecular weight fibroblast growth factor-2. Dev. Dyn.

[b7] Chandraratna PA, Aronow MS, Aronow WS (1982). Significance of echocardiographic left atrial enlargement in aortic stenosis. Clin. Cardiol.

[b8] Coffin JD, Florkiewicz RZ, Neumann J, Mort-Hopkins T (1995). Abnormal bone growth and selective translational regulation in basic fibroblast growth factor (FGF-2) transgenic mice. Mol. Biol. Cell.

[b9] Colan SD, Borow KM, Neumann A (1984). Left ventricular end-systolic wall stress-velocity of fiber shortening relation: a load-independent index of myocardial contractility. J. Am. Coll. Cardiol.

[b10] Dealmeida A, Sedmera D (2009). Fibroblast Growth Factor-2 regulates proliferation of cardiac myocytes in normal and hypoplastic left ventricles in the developing chick. Cardiol. Young.

[b11] Devereux RB, Reichek N (1977). Echocardiographic determination of left ventricular mass in man. Anatomic validation of the method. Circulation.

[b12] Dono R, Texido G, Dussel R, Ehmke H, Zeller R (1998). Impaired cerebral cortex development and blood pressure regulation in FGF-2-deficient mice. EMBO J.

[b13] Du XJ (2004). Gender modulates cardiac phenotype development in genetically modified mice. Cardiovasc. Res.

[b14] Evangelista A, Flachskampf FA, Erbel R, Antonini-Canterin F, Vlachopoulos C, Rocchi G (2010). Echocardiography in aortic diseases: EAE recommendations for clinical practice. Eur. J. Echocardiogr.

[b15] Finsen AV, Christensen G, Sjaastad I (2005). Echocardiographic parameters discriminating myocardial infarction with pulmonary congestion from myocardial infarction without congestion in the mouse. J. Appl. Physiol.

[b16] Garcia MJ, Thomas JD, Klein AL (1998). New Doppler echocardiographic applications for the study of diastolic function. J. Am. Coll. Cardiol.

[b17] Garmy-Susini B, Delmas E, Gourdy P, Zhou M, Bossard C, Bugler B (2004). Role of fibroblast growth factor-2 isoforms in the effect of estradiol on endothelial cell migration and proliferation. Circ. Res.

[b18] Gentile F, Ornaghi M, Esposti D, Triulzi MO (1993). Hemodynamic effects of nifedipine in patients with asymptomatic aortic regurgitation: evaluation by Doppler echocardiography. Acta Cardiol.

[b19] Holzenberger M, Hamard G, Zaoui R, Leneuve P, Ducos B, Beccavin C (2001). Experimental IGF-I receptor deficiency generates a sexually dimorphic pattern of organ-specific growth deficits in mice, affecting fat tissue in particular. Endocrinology.

[b20] House SL, Branch K, Newman G, Doetschman T, Schultz JJ (2005). Cardioprotection induced by cardiac-specific overexpression of fibroblast growth factor-2 is mediated by the MAPK cascade. Am. J. Physiol. Heart Circ. Physiol.

[b21] House SL, Melhorn SJ, Newman G, Doetschman T, Schultz JJ (2007). The protein kinase C pathway mediates cardioprotection induced by cardiac-specific overexpression of fibroblast growth factor-2. Am. J. Physiol. Heart Circ. Physiol.

[b22] House SL, House BE, Glascock B, Kimball T, Nusayr E, Schultz JE (2010). Fibroblast growth factor 2 mediates isoproterenol-induced cardiac hypertrophy through activation of the extracellular regulated kinase. Mol. Cell. Pharmacol.

[b23] Itoh N, Ornitz DM (2011). Fibroblast growth factors: from molecular evolution to roles in development, metabolism and disease. J. Biochem.

[b24] Kuroda T, Shiina A, Suzuki O, Fujita T, Noda T, Tsuchiya M (1989). Prediction of prognosis of patients with idiopathic dilated cardiomyopathy: a comparison of echocardiography with cardiac catheterization. Jpn. J. Med.

[b25] Lavine KJ, Ornitz DM (2008). Fibroblast growth factors and Hedgehogs: at the heart of the epicardial signaling center. Trends Genet.

[b26] Liao S, Porter D, Scott A, Newman G, Doetschman T, Schultz JJ (2007). The cardioprotective effect of the low molecular weight isoform of fibroblast growth factor-2: the role of JNK signaling. J. Mol. Cell. Cardiol.

[b27] Liao S, Bodmer J, Pietras D, Azhar M, Doetschman T, Schultz JJ (2009). Biological functions of the low and high molecular weight protein isoforms of fibroblast growth factor-2 in cardiovascular development and disease. Dev. Dyn.

[b28] Liao S, Bodmer JR, Azhar M, Newman G, Coffin JD, Doetschman T (2010). The influence of FGF2 high molecular weight (HMW) isoforms in the development of cardiac ischemia-reperfusion injury. J. Mol. Cell. Cardiol.

[b29] McEniery CM, Yasmin, Wallace S, Maki-Petaja K, McDonnell B, Sharman JE (2005). Increased stroke volume and aortic stiffness contribute to isolated systolic hypertension in young adults. Hypertension.

[b30] Montero A, Okada Y, Tomita M, Ito M, Tsurukami H, Nakamura T (2000). Disruption of the fibroblast growth factor-2 gene results in decreased bone mass and bone formation. J. Clin. Invest.

[b31] Neagoe C, Opitz CA, Makarenko I, Linke WA (2003). Gigantic variety: expression patterns of titin isoforms in striated muscles and consequences for myofibrillar passive stiffness. J. Muscle Res. Cell Motil.

[b32] Oh JK, Appleton CP, Hatle LK, Nishimura RA, Seward JB, Tajik AJ (1997). The noninvasive assessment of left ventricular diastolic function with two-dimensional and Doppler echocardiography. J. Am. Soc. Echocardiogr.

[b33] Ortega S, Ittmann M, Tsang SH, Ehrlich M, Basilico C (1998). Neuronal defects and delayed wound healing in mice lacking fibroblast growth factor 2. Proc. Natl. Acad. Sci. USA.

[b34] Pellieux C, Foletti A, Peduto G, Aubert JF, Nussberger J, Beermann F (2001). Dilated cardiomyopathy and impaired cardiac hypertrophic response to angiotensin II in mice lacking FGF-2. J. Clin. Invest.

[b35] Prats H, Kaghad M, Prats AC, Klagsbrun M, Lelias JM, Liauzun P (1989). High molecular mass forms of basic fibroblast growth factor are initiated by alternative CUG codons. Proc. Natl. Acad. Sci. USA.

[b36] Rottman JN, Ni G, Brown M (2007). Echocardiographic evaluation of ventricular function in mice. Echocardiography.

[b37] Schellings MW, Pinto YM, Heymans S (2004). Matricellular proteins in the heart: possible role during stress and remodeling. Cardiovasc. Res.

[b38] Schneider MD, Parker TG (1991). Cardiac growth factors. Prog. Growth Factor Res.

[b39] Schultz JE, Witt SA, Nieman ML, Reiser PJ, Engle SJ, Zhou M (1999). Fibroblast growth factor-2 mediates pressure-induced hypertrophic response. J. Clin. Invest.

[b40] Smith VE, White WB, Karimeddini MK (1987). Echocardiographic assessment of left ventricular diastolic performance in hypertensive subjects. Correlation with changes in left ventricular mass. Hypertension.

[b41] Tanaka N, Dalton N, Mao L, Rockman HA, Peterson KL, Gottshall KR (1996). Transthoracic echocardiography in models of cardiac disease in the mouse. Circulation.

[b42] Touriol C, Bornes S, Bonnal S, Audigier S, Prats H, Prats AC (2003). Generation of protein isoform diversity by alternative initiation of translation at non-AUG codons. Biol. Cell.

[b43] Tsang TS, Barnes ME, Gersh BJ, Bailey KR, Seward JB (2002). Left atrial volume as a morphophysiologic expression of left ventricular diastolic dysfunction and relation to cardiovascular risk burden. Am. J. Cardiol.

[b44] Vitarelli A, Gheorghiade M (1998). Diastolic heart failure: standard Doppler approach and beyond. Am. J. Cardiol.

[b45] Wallace SM, Yasmin, McEniery CM, Maki-Petaja KM, Booth AD, Cockcroft JR (2007). Isolated systolic hypertension is characterized by increased aortic stiffness and endothelial dysfunction. Hypertension.

[b46] Watkins BP, Bolender DL, Lough J, Kolesari GL (1998). Teratogenic effects of implanting fibroblast growth factor-2-soaked beads in the cardiac region of the stage 24 chick embryo. Teratology.

[b47] Xiao L, Liu P, Li X, Doetschman T, Coffin JD, Drissi H (2009). Exported 18-kDa isoform of fibroblast growth factor-2 is a critical determinant of bone mass in mice. J. Biol. Chem.

[b48] Yellin EL, Nikolic S, Frater RW (1990). Left ventricular filling dynamics and diastolic function. Prog. Cardiovasc. Dis.

[b49] Yu PJ, Ferrari G, Galloway AC, Mignatti P, Pintucci G (2007). Basic fibroblast growth factor (FGF-2): the high molecular weight forms come of age. J. Cell. Biochem.

[b50] Zhou M, Sutliff RL, Paul RJ, Lorenz JN, Hoying JB, Haudenschild CC (1998). Fibroblast growth factor 2 control of vascular tone. Nat. Med.

[b51] Zile MR, Brutsaert DL (2002). New concepts in diastolic dysfunction and diastolic heart failure: Part I: diagnosis, prognosis, and measurements of diastolic function. Circulation.

[b52] Zile MR, Baicu CF, Bonnema DD (2005). Diastolic heart failure: definitions and terminology. Prog. Cardiovasc. Dis.

